# Aging Properties of Phenol-Formaldehyde Resin Modified by Bio-Oil Using UV Weathering

**DOI:** 10.3390/polym10111183

**Published:** 2018-10-24

**Authors:** Yuxiang Yu, Pingping Xu, Miaomiao Chang, Jianmin Chang

**Affiliations:** College of Materials Science and Technology, Beijing Forestry University, 35 Qinghua East Road, Haidian District, Beijing 100083, China; yuyuxiang0612@163.com (Y.Y); m18813102213@163.com (P.X.); maoerdan0615@163.com (M.C.)

**Keywords:** aging properties, phenol-formaldehyde resin, bio-oil, UV weathering

## Abstract

The aging properties of phenol-formaldehyde resin modified by bio-oil (BPF) were analyzed using ultraviolet (UV) weathering. The variations on bonding strength of BPF were measured, and the changes on microstructure, atomic composition and chemical structure of BPF were characterized by using a scanning electron microscope (SEM), X-ray photoelectron spectroscopy (XPS) and nuclear magnetic resonance (NMR), respectively. With the increase of aging time, the bonding strength decreased gradually, the resin surface became rougher and the O/C radio of resin surface increased. However, the loss rate of bonding strength of BPFs was 9.6–23.0% lower than that of phenol-formaldehyde resin (PF) after aging 960 h. The aging degree of BPF surfaces was smaller in comparison to PF at the same aging time. These results showed that the bio-oil had a positive effect on the anti-aging property. Analytical results revealed that with increasing the aging time, the XPS peak area of C–C/C–H decreased, while that of C=O and O–C=O increased. The intensity of methylene and ether bridges in NMR analysis decreased along with increasing the intensity of aldehydes, ketones, acids and esters. These results indicated that the aging mechanism of BPF was a process of the breakage of molecular chains and formation of oxygen-containing compounds.

## 1. Introduction

Phenol-formaldehyde resin (PF) has been widely used in outdoor wooden products because of its excellent mechanical properties, thermal stability and electrical insulation [1,2,3]. However, the environmental factors (light, heat, oxygen, water, etc.) accelerate the aging of PF and further shorten the service life of outdoor wood products [4,5]. The aging of PF highly depends on its molecular structure [4,6,7,8]. For example, the phenolic hydroxyl and methylene groups in the molecular chain of PF are easy to be oxidized [6,7]. A large number of hydrophilic phenolic hydroxyl and hydroxymethyl groups of PF easily absorb atmospheric moisture, resulting in the damage of the glue line and hydrolysis of resin [8].

The modification of anti-aging property for PF is mainly achieved by three methods. One is to reduce the number of active groups, such as phenolic groups and hydroxymethyl groups, in the molecular chain of PF. Another is to protect the weak links of phenolic groups and methylene groups to reduce the possibility of oxidation and hydrolysis. The third way is to introduce anti-aging groups into the chemical structure of PF. A number of studies found that the introduction of inorganic element like boron [9,10] and titanium [11] in the synthesis of resin effectively improved the stability and water resistance of the resin. These inorganic compounds tend to form covalent bond with phenolic hydroxyl or hydroxymethyl groups of PF and the bond energy of B–O and Ti–O is much higher than that of C–C bond, thus improving the thermal stability. Meanwhile, the reduction of hydroxyl groups improves the water resistance. Adding nanometer material, such as nano montmorillonite [12,13] and nanofibril [5] in resin, could form a connection interface around the polar molecular chain and enhance the intermolecular forces between molecular chain, thus improving the stability and toughness of resin. In addition, using modifiers like carbazole [14,15] and cashew nut shell liquid [16] to introduce heat-resistant aromatic heterocyclic structures or hydrophobic groups into molecular chain of resin, enhances the anti-aging of resin.

Bio-oil, a liquid produced by fast pyrolysis and condensation of renewable biomass, is rich in phenols, aldehydes, esters and other organic substances [17,18,19]. Bio-oil can be directly used as fuel oil, or separated and purified to prepare chemical products [18,19]. Many researchers have successfully used bio-oil to partially replace phenol to synthesize the bio-oil phenol-formaldehyde resin (BPF) [20,21,22,23,24]. Aslan et al. [22] used bio-oil to substitute the phenol, and found that the 10 wt.% bio-oil modified PF had a similar molecular structure and higher tensile-shear strength to commercial PF. Chaouch et al. [24] showed that the BPF whose substitution degree was up to 50%, presented a nice storage stability and thermal stability, and good shear strength comparable to PF. Additionally, the long-chain unsaturated hydrocarbons and esters in bio-oil have the potential to improve the toughness and water resistance of resin. At present, researches on BPF mainly focus on the synthesis process and performance characterization. However, to the best of our knowledge, little literature seems to be available on the aging properties of BPF.

As an adhesive for outdoor wooden products, it is quite necessary to study the aging properties of BPF. Therefore, in this study, the aging properties of BPFs with different substitute rates of bio-oil to phenol (B/P substitute rates) were studied using ultraviolet (UV) weathering. The variations of bonding strength of plywood and microscopic structure of BPF films during aging were examined. The changes on O/C element radio and functional groups in the film surface of BPF during aging were analyzed by X-ray photoelectron spectroscopy (XPS). The chemical structure changes of resin film were further estimated by means of solid nuclear magnetic resonance (NMR) to clarify the aging behavior of BPF.

## 2. Materials and Methods

### 2.1. Materials

Bio-oil, an acid liquid (pH 3.5), was obtained by fast pyrolysis of *Larix gmelinii* (Rupr.) Kuzen in a fluidized bed at 550 °C for 2–3 s by the Laboratory of Fast Pyrolysis of Biomass and Productive Utilization (Beijing Forestry University, Beijing, China). The bio-oil was composed of 25.4% phenols, 12.6% ketones, 7.9% aldehydes, 6.6% organic acids, 4.8% esters, 4.2% alcohols, 29.8% water and 8.7% other compounds. Phenol, formaldehyde (aqueous solution, 37 wt.%) and sodium hydroxide (NaOH) were purchased from Xilong Chemical Reagent Co., Ltd., Guangdong, China. Poplar veneers (400 mm × 400 mm × 1.5 mm, 8% moisture content) were provided by Xinda wooden Co., Ltd., Hebei, China.

### 2.2. Synthesis and Characterization of Resins

The BPF with different B/P substitute rates (0, 10, 20, and 30 wt.%) were synthesized in a three-neck flask. The molar ratio of phenol (include bio-oil)/formaldehyde/NaOH was 1:2:0.5. In the first step, phenol, 75% of total formaldehyde (37 wt.%) and NaOH solution (30 wt.%) were added to the flask. Then this system was heated to 90 °C between 20 and 30 min, and held for 40 min. After this step, the temperature was dropped to 80 °C, and the residual 25% of total formaldehyde (37 wt.%) and NaOH solution (30 wt.%), and bio-oil were added. Then, the system was heated to 90 °C and held for 30 min. Finally, the system was cooled down to 40 °C in 20 min to yield resin. These prepared resins were denoted as PF, 10%BPF, 20%BPF and 30%BPF.

The pH, viscosity and solid content of resin were measured on the basis of Chinese standards (GB/T 14074-2013). The water absorption of resin was evaluated according to Chinese standards (GB/T 1034-2008). In this test, the resin film (80 mm × 10 mm × 2 mm) was put in a drying oven at 108 °C for 1 day. Next, the film was dipped into distilled water at 25 °C for 1 day, and then the value was computed. Each test above was repeated at least in three times. The characteristics of BPFs and PF are tabulated in Table 1.

### 2.3. Preparation of Aging Materials

The three-layer plywood was prepared with the synthesized resin. Wheat flour (10 wt.% of resin) was added as filler in the resin. Firstly, each side of the core veneer was coated with 200–220 g/m^2^ resin. Secondly, the three veneers (the coated veneer was placed between two uncoated veneers) were hot-pressed at 140 °C under 1.1 MPa for 6.5 min. Finally, the plywood was cut to prepare the small aging plywood specimen (100 mm × 25 mm × 3 mm). The resin film was cured by a stainless steel mold (80 mm × 10 mm × 2 mm) in a dry oven at 80 °C for 4 h and then at 140 °C for 1 h. The plywood specimen and resin films were put in a chamber (23 °C, 55% relative humidity) for 2 days before UV weathering.

### 2.4. UV Weathering

The plywood samples and resin films were examined by a UV accelerated weathering tester (Yiheng Co., Ltd., Shanghai, China) according to the ASTM G 154. Each 12 h weathering cycle consisted of 8 h of UV exposure at 60 °C and 4 h condensation at 50 °C.

### 2.5. Analysis

The bonding strength of the plywood was evaluated on the basis of Chinese standard (GB/T 17657-2013). The scanning electron microscope (SEM) analysis of resin film was measured by SU8010 SEM (Hitachi, Tokyo, Japan). The X-ray photoelectron spectroscopy (XPS) analysis of the resin film was conducted by PHI Quantera SXM (ULVAC-PHI, Chigasaki, Japan). The solid state nuclear magnetic resonance (NMR) analysis of the resin film was evaluated by JNM-ECZ600R (JEOL, Tokyo, Japan).

## 3. Results and Discussion

### 3.1. Bonding Strength

**Figure 1:**
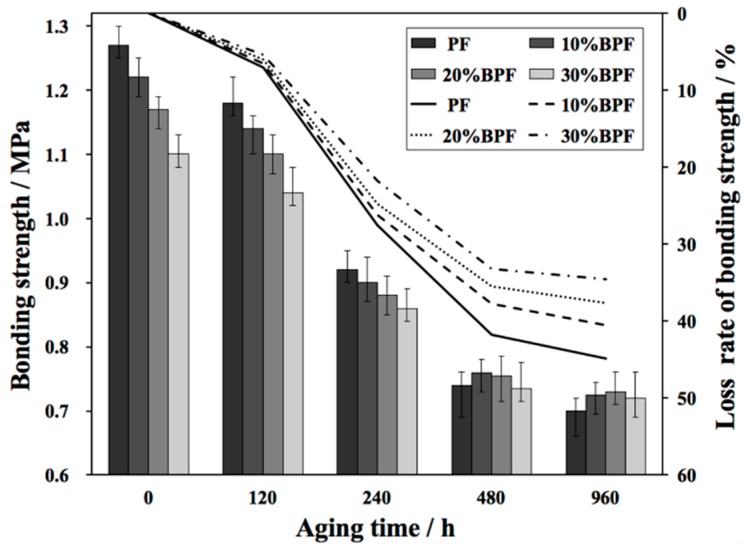
Bonding strength of plywood bonded with BPFs and PF during ultraviolet (UV) weathering.

The changes on the bonding strength of plywood with BPFs and PF during UV weathering are shown in Figure 1. As can be seen, the changes of bonding strength could be divided into three stages prolonged aging time: initial stage (0–120 h), middle stage (120–480 h) and last stage (480–960 h). The decrease of bonding strength in the initial stage was mainly related to the hydrolysis of resin and plywood, as well as the plasticization and swelling of resin [5,8], thus leading to the interfacial debonding. This was because water was easier to enter into the resin than UV through the hydrophilic groups in resin and plywood. However, the descending speed of the initial stage was smaller than that of the middle stage, owing to the post-curing of resin in the early stage of aging. This corresponded to that of Strzemiecka et al. [25], who outlined that the early chemical changes of cured PF during storage were mainly the result of post-curing reaction. Meanwhile, with increasing the aging time, the degradation resulted from water provided more possibilities for the aging caused by the combination of UV, heat, water and oxygen, resulting in the rapid decline of bonding strength in the middle stage. The decreasing rate of bonding strength became slower in the last stage because the residual groups and chemical bonds were more stable to resist the aging from the environment.

Compared with PF plywood, the bonding strength of BPF plywood decreased, and the descent degree of bonding strength was gradually apparent with the increase of B/P substitute rate. This happened because the reactivity of the phenol compounds in bio-oil to formaldehyde was lower than that of phenol, leading to the decrease of polymerization degree. This had been discussed by Aslan et al. [22] and Ozbay and Ayrilmis [26]. Additionally, Ozbay and Ayrilmis [26] indicated another reason for the decrease of bonding strength was the increasing viscosity of resin by adding bio-oil (Table 1), which made the resin harder to penetrate into veneers to form nails. Besides, the decrease of pH value with the addition of bio-oil (Table 1) might have the negative influence of bonging strength [27]. However, the decreasing rate of bonding strength became slower with increasing the B/P substitute rate. After aging 960 h, the loss rate of bonding strength for plywood with BPFs were 40.6%, 37.6% and 35.5%, respectively, which were 9.6–23.0% lower than that with PF (44.9%). This suggested that the anti-aging property of resin improved after adding bio-oil. One possible reason was the lower water absorbance of BPF (Table 1), which weakened the aging effort of water. Another possible reason was that the substances like aldehydes in bio-oil reacted with the unreacted phenol hydroxyl and hydroxymethyl groups, which reduced the possibility of hydrolysis and thermal oxygen degradation of the resin.

### 3.2. Microstructure

**Figure 2:**
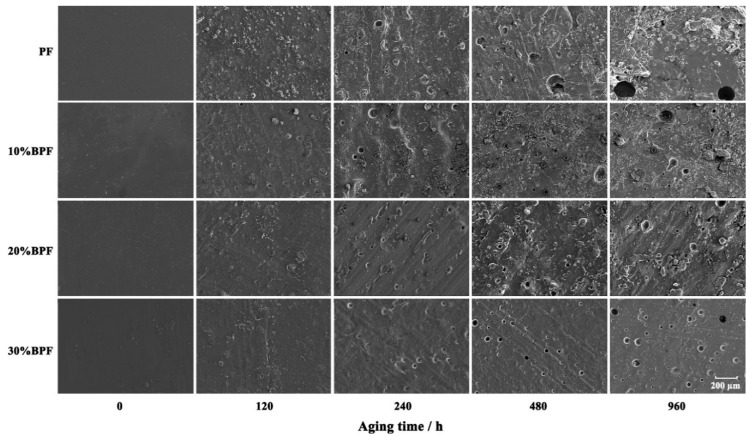
Scanning electron microscope (SEM) images of BPF and PF films during UV weathering.

Figure 2 shows the changes of microstructure of cured BPF and PF films during UV weathering. The unaged resin films had smooth and flat surface structure. After aging 120 h, the smoothness of the resin films decreased, and tiny particles appeared on the surfaces. After 240 h of aging, the surface roughness of the resin films further increased and both the number and size of particles increased. At the same time, some tiny holes appeared on the resin film surfaces, which meant that water and light could pass through these tiny holes into the inside of the resin, accelerating the aging of the resin. As the aging process continued, the diameter of the hole increased gradually, and finally, cracks appeared around the hole.

As seen in Figure 2, at the same aging time, the aging degree of BPF film surfaces were smaller than that of PF. When aging 960 h, the hole size in BPF film surfaces were smaller compared with PF, indicating that the adding of bio-oil improved the anti-aging property of resin. These might be due to the lower water absorbance of BPF. In addition, the improvement effect was more obvious with the increase of B/P substitute rate, which had a similar change trend with the bonding strength in Section 3.1.

### 3.3. XPS Analysis

**Figure 3:**
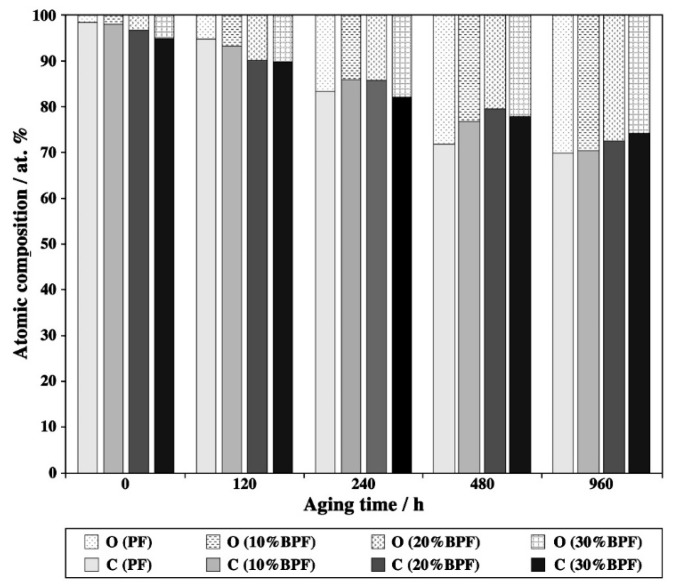
X-ray photoelectron spectroscopy (XPS) surface atomic composition (at.%) of BPF and PF films during UV weathering.

XPS can give information about the changes of atomic composition and chemical bonds of resin film surface during aging. The changes of XPS surface atomic composition of BPF and PF films during UV weathering are shown in Figure 3. From Figure 3, it could be found that the surface atomic composition of resins was highly affected by the aging time. The proportion of C dropped dramatically along with gradually increasing that of O on the resin film surface as the progress of aging time. This means that the O/C ratio of the resin surface increased, indicating that the aging progress caused by UV weathering included the oxidation aging process. Compared to PF, the initial O/C radio of BPF films were larger, which was due to the abundant oxygenated substances of bio-oil. However, adding bio-oil reduced the oxidation effect of UV weathering. After aging 960 h, the C/O radio of PF, 10%BPF, 20%BPF and 30%BPF films were 0.432, 0.420, 0.379 and 0.348, respectively. The decreasing rate of C/O radio of BPF film was 21.4–74.2% less than that of PF film.

**Figure 4:**
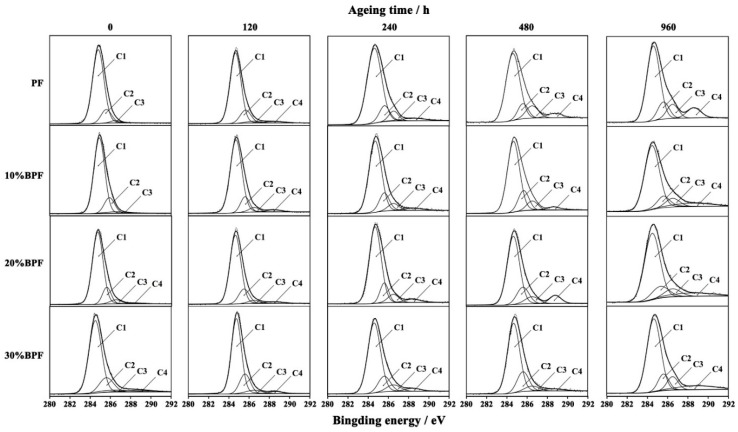
XPS C 1s spectra of cured BPF and PF films during UV weathering.

**Figure 5:**
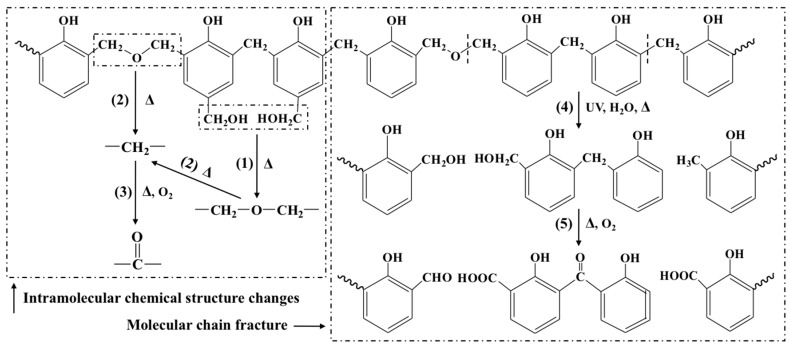
The schematic representation on the aging of BPF and PF in case of UV weathering.

The high resolutions of the XPS C 1s spectra of cured BPF and PF films during UV weathering are shown in Figure 4 and quantification of chemical state curve fitting are listed in Table 2 [28]. With the prolonging of aging time, the peak area of C–C/C–H at 284.8 eV decreased because of the oxidation on the resin surface by aging. The peak area of C–O at 285.6 eV first increased and then decreased, suggesting that some substances with C–O bond were the intermediates of aging and could be further oxidized to form other oxygen-containing groups, such as aldehyde groups and carboxyl groups. These could be proved by the changes on the peak at 286.5 and 288.4 eV, which were attributed to the C=O and O–C=O, respectively. The initial peak areas of C=O and O–C=O before aging were relatively low; however, these increased after UV weathering. This might be due to the production of small molecule compounds by the breakage of the ether bridge and oxidation of methylene bridge in the molecular chain of resins. Guo et al. [6] studied the thermos-oxidative aging at low temperature and found that the oxidation of methylene bridges occurred at 60 °C (the temperature of UV weathering was 60 °C). Therefore, based on these results, it is possible to propose the aging mechanism of BPF and PF, which is depicted in Figure 5.

Compared to PF, the initial peak area of C–C/C–H from BPFs were smaller, while that of C=O and C–C=O were larger, which means that the oxygenated substances in bio-oil were successfully involved in the synthesis of resins, leading to the increase of the amount of C=O and C–C=O groups. However, after aging 960 h, the peak area of C–C/C–H from BPFs were larger, and that of C=O and C–C=O from BPFs were smaller, indicating that the oxidation degree of BPFs were lower. This means that the bio-oil had a positive effect on the anti-aging property of PF, which was quite consistent with the results shown in Section 3.1 and Section 3.2.

### 3.4. NMR Analysis

**Figure 6:**
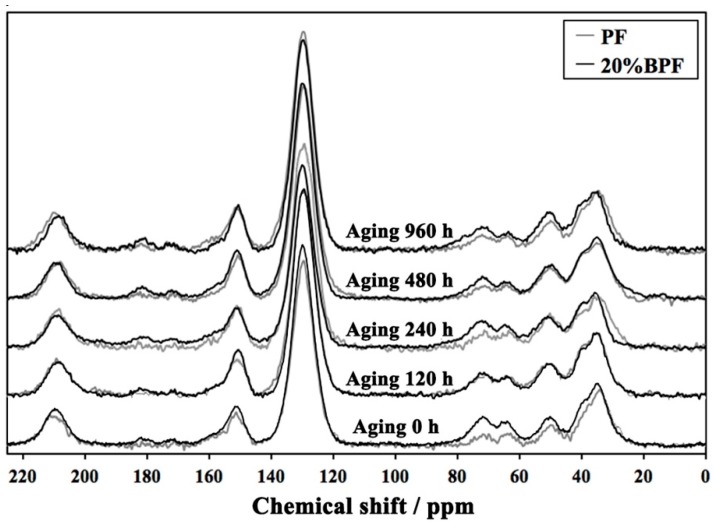
Nuclear magnetic resonance (NMR) analysis of cure 20%BPF and PF during UV weathering.

In order to further determine the chemical structure changes and compare the differences between BPF and PF films during UV weathering, the comparison of ^13^C NMR spectra of resin films are shown in Figure 6, and the chemical structure change of resin films during UV weathering were displayed in Table 3 [9,11,29]. The integral of phenolic carbon at about 150 ppm was selected as normalization and its intensity was set to 1.00. For instance, the ratio of integral value of 210 ppm/150 ppm was represented the degree of C=O groups from aldehydes or ketones.

Compared to PF, the peaks of carboxylic acid (186–176 ppm) and carboxylic ester (176–168 ppm) are more obvious. This was related to the addition of bio-oil in the synthesis of resin, which introduced much organic carboxylic acids and esters into the resin. Moreover, the intensity of methylene bridges (45–29 ppm) and dimethylene ether bridges (c: 75–65 ppm) both increased after adding bio-oil. This might be due to: (1) the facilitation of bio-oil to the polycondensation of resin [29]; (2) the abundance of substance with methylene and ether groups in bio-oil.

After aging 120 h, the intensity of methylene and dimethylene ether bridges increased, which was due to the post-curing of resin. At the same time, the post-curing of resin also increased the intensity of substituted ortho and para aromatic carbons (137–123 ppm) and decreased that of methylol groups (63–58 ppm). Possible reactions are shown in Figure 5(1),(2). After 960 h of aging, the intensity of aldehydes and ketones (220–200 ppm), carboxylic acids and esters increased, while that of methylene and ether bridges decreased. These indicated that the methylene and ether bridges in the resin broke to form a series of oxygen-containing compounds like aldehydes, ketones, acids and esters. Possible reactions are shown in Figure 5(4),(5). Compared to PF, the reduction of the methylene and ether bridges from 20%BPF was lower, indicating that the BPF had better anti-aging properties.

## 4. Conclusions

The aging properties of BPF were investigated using UV weathering. With the increase of aging time, the bonding strength, microstructure and surface atomic composition of BPF changed significantly. This was mainly due to the variation in the chemical structure of BPF during aging, including the reactions among molecular chains, such as post-curing and the fracture of molecular chain to form small molecule compounds. In addition, adding bio-oil into PF improved the anti-aging property. The decreasing rate of bonding strength, the aging degree on the microstructure and the decreasing rate of C/O radio of BPF were smaller than those of PF. These results are related to the impact of bio-oil on the variation chemical structure of resin after aging. After aging 960 h, the XPS peak area of C–C/C–H from BPF were larger and the peak areas of C=O and C–C=O from BPF were smaller than those of PF resin. Furthermore, compared to PF, the reduction of the methylene and ether bridges of BPF in NMR analysis was lower.

## Figures and Tables

**Table 1:** Characteristics of bio-oil phenol-formaldehyde resins (BPFs) and phenol-formaldehyde resin (PF).

Resins	B/P Substitute Rate (%)	Characteristics			
		pH (25 °C)	Viscosity (25 °C, mPa·s)	Solids Content (%)	Water Absorption (25 °C, 24 h, %)
PF	0	10.98 ± 0.12	117 ± 22	47.64 ± 0.28	30.26 ± 1.12
10%BPF	10	10.57 ± 0.15	185 ± 40	46.42 ± 0.19	27.34 ± 0.98
20%BPF	20	10.27 ± 0.09	278 ± 31	45.23 ± 0.23	24.33 ± 0.73
30%BPF	30	9.95 ± 0.16	636 ± 58	42.69 ± 0.21	22.32 ± 1.36

**Table 2:** XPS peak fitting date from the high resolution C 1s spectra of BPF and PF films during UV weathering.

Aging Time	Peak Area (%)															
	C1 (284.8, C–H) ^a^				C2 (285.6, C–O)				C3 (286.5, C=O)				C4 (288.4, O–C=O)			
	0	1	2	3 ^b^	0	1	2	3	0	1	2	3	0	1	2	3
0	84.3	81.9	80.0	78.6	13.6	14.6	14.2	14.8	2.1	3.0	4.8	5.2	—	0.5	1.0	1.4
120	80.3	79.8	77.9	74.0	14.2	12.8	15.3	15.1	3.1	4.7	3.7	7.3	2.4	2.7	3.1	3.6
240	75.1	73.2	74.3	72.3	14.8	16.5	14.4	18.0	5.6	6.6	6.7	5.5	4.5	3.7	4.6	4.2
480	70.9	70.3	70.9	71.2	13.9	16.9	16.0	17.4	10.0	8.2	6.6	6.3	5.2	4.6	6.5	5.1
960	66.2	67.0	68.1	68.4	13.4	14.7	14.2	14.9	10.8	8.7	8.6	8.2	9.6	9.6	9.1	8.5

^a^ Element group (Binding energy (eV), Bond type); ^b^ Resin type (0: PF; 1: 10%BPF; 2: 20%BPF; 3: 30%BPF).

**Table 3:** Solid-state ^13^C NMR assignment and quantitative analysis of chemical groups for cured 20%BPF and PF during UV weathering.

Chemical Group Structure	Chemical Shirt (ppm)	Calculation									
		20%BPF					PF				
		0	120	240	480	960	0	120	240	480	960
C=O form aldehydes/ketones	220–200	0.98	1.08	1.11	1.19	1.24	0.85	1.10	1.26	1.37	1.32
C=O from carboxylic acids	186–176	0.09	0.14	0.23	0.26	0.30	0.02	0.02	0.03	0.12	0.18
C=O from esters	176–168	0.05	0.05	0.12	0.17	0.16	0.01	0.01	0.02	0.09	0.14
Phenolic carbon	160–148	1.00	1.00	1.00	1.00	1.00	1.00	1.00	1.00	1.00	1.00
Substituted ortho and para aromatic carbons	137–123	4.19	4.52	4.29	4.26	4.20	4.21	4.43	4.31	4.22	4.07
Dimethylene ether bridges	75–65	0.56	0.68	0.71	0.50	0.48	0.20	0.46	0.26	0.18	0.24
Methylol groups	63–58	0.32	0.54	0.43	0.33	0.27	0.19	0.36	0.25	0.20	0.23
Benzylamines	58–45	0.53	0.88	0.91	1.11	1.20	0.56	1.06	0.89	0.96	0.74
Methylene bridges	45–29	2.27	2.40	2.36	2.27	2.10	2.17	2.38	2.29	2.16	2.03
